# Genetic Factors Associated with Exercise Performance in Atmospheric Hypoxia

**DOI:** 10.1007/s40279-015-0309-8

**Published:** 2015-02-15

**Authors:** Philip J. Hennis, Alasdair F. O’Doherty, Denny Z. H. Levett, Michael P. W. Grocott, Hugh M. Montgomery

**Affiliations:** 1 UCLH NIHR Biomedical Research Centre, Institute of Sport and Exercise Health, University College London Centre for Altitude Space and Extreme Environment Medicine, 170 Tottenham Court Road, London, W1T 7HA UK; 2 Department of Sport, Health and Exercise Science, University of Hull, Hull, UK; 3 Integrative Physiology and Critical Illness Group, Clinical and Experimental Sciences, Sir Henry Wellcome Laboratories, Faculty of Medicine, University of Southampton, University Hospital Southampton NHS Foundation Trust, Southampton, UK; 4 Anaesthesia and Critical Care Research Unit, University Hospital Southampton NHS Foundation Trust, Southampton, UK; 5 NIHR Southampton Respiratory Biomedical Research Unit, Southampton, UK

## Abstract

**Background and Objective:**

‘Natural selection’ has been shown to have enriched the genomes of high-altitude native populations with genetic variants of advantage in this hostile hypoxic environment. In lowlanders who ascend to altitude, genetic factors may also contribute to the substantial interindividual variation in exercise performance noted at altitude. We performed a systematic literature review to identify genetic variants of possible influence on human hypoxic exercise performance, commenting on the strength of any identified associations.

**Criteria for considering studies for this review:**

All studies of the association of genetic factors with human hypoxic exercise performance, whether at sea level using ‘nitrogen dilution of oxygen’ (normobaric hypoxia), or at altitude or in low-pressure chambers (field or chamber hypobaric hypoxia, respectively) were sought for review.

**Search strategy for identification of studies:**

Two electronic databases were searched (Ovid MEDLINE, Embase) up to 31 January 2014. We also searched the reference lists of relevant articles for eligible studies. All studies published in English were included, as were studies in any language for which the abstract was available in English.

**Data collection and analysis:**

Studies were selected and data extracted independently by two reviewers. Differences regarding study inclusion were resolved through discussion. The quality of each study was assessed using a scoring system based on published guidelines for conducting and reporting genetic association studies.

**Results:**

A total of 11 studies met all inclusion criteria and were included in the review. Subject numbers ranged from 20 to 1,931 and consisted of healthy individuals in all cases. The maximum altitude of exposure ranged from 2,690 to 8,848 m. The exercise performance phenotypes assessed were mountaineering performance (*n* = 5), running performance (*n* = 2), and maximum oxygen consumption ($\dot{V}$O_2_max) (*n* = 4). In total, 13 genetic polymorphisms were studied, four of which were associated with hypoxic exercise performance. The adenosine monophosphate deaminase (AMPD1) C34T (rs17602729), beta2-adrenergic receptor (ADRB2) Gly16Arg single nucleotide polymorphism (SNP) (rs1042713), and androgen receptor CAG repeat polymorphisms were associated with altitude performance in one study, and the angiotensin I-converting enzyme (ACE) insertion/deletion (I/D) (rs4646994) polymorphism was associated with performance in three studies. The median score achieved in the study quality analysis was 6 out of 10 for case–control studies, 8 out of 10 for cohort studies with a discrete outcome, 6 out of 9 for cohort studies with a continuous outcome, and 4.5 out of 8 for genetic admixture studies.

**Conclusion:**

The small number of articles identified in the current review and the limited number of polymorphisms studied in total highlights that the influence of genetic factors on exercise performance in hypoxia has not been studied in depth, which precludes firm conclusions being drawn. Support for the association between the ACE-I allele and improved high-altitude performance was the strongest, with three studies identifying a relationship. Analysis of study quality highlights the need for future studies in this field to improve the conduct and reporting of genetic association studies.

**Electronic supplementary material:**

The online version of this article (doi:10.1007/s40279-015-0309-8) contains supplementary material, which is available to authorized users.

## Key Points

**Table:** 

The size and scope of the literature regarding the role of genetics on high-altitude exercise performance is limited.
The association between the angiotensin I-converting enzyme (ACE) insertion (I) allele and improved high-altitude performance has the strongest support.
The volume and quality of the literature limits firm conclusions being made and needs to be addressed in future studies.

## Introduction

With ascent to altitude, barometric pressure falls, and with it the partial pressure of inspired oxygen (PiO_2_). Despite diverse adaptive responses, the arterial partial pressure of oxygen (PaO_2_) also falls. Such ‘hypobaric hypoxia’ impairs physical performance and can cause pathology (acute mountain sickness [AMS]), which may be fatal (e.g. high-altitude pulmonary oedema [HAPE] or high-altitude cerebral oedema [HACE]).

Longstanding communities of high-altitude residents exist worldwide, most notably on the Tibetan Plateau and Andean Altiplano, but also in the area of the Great Rift Valley in Ethiopia. ‘Natural selection’ has enriched the genomes of high-altitude native populations, conferring advantage in this hostile hypoxic environment [1]. Meanwhile, increasing numbers of native lowlanders travel to high altitudes for recreation or work. Amongst these lowland populations, similar genetic factors may contribute to the substantial inter-individual variation in exercise performance and wellbeing observed at altitude [2, 3]. Whilst attempts have been made to identify genetic variants associated with improved high-altitude performance, the results of such studies have yet to be collated. We thus set out to systematically review the published literature and identify genetic variants of influence on human hypoxic exercise performance, commenting on the strength of any identified associations.

## Methods

### Search Strategy

#### Criteria for Considering Studies for this Review

All studies of the association of genetic factors with human hypoxic exercise performance, whether at sea level using ‘nitrogen dilution of oxygen’ (normobaric hypoxia), or at altitude or in low-pressure chambers (field or chamber hypobaric hypoxia, respectively) were sought for review. ‘Altitude’ was defined as a real or simulated (normobaric hypoxia) elevation of ≥2,000 m above sea level. Performance measures were defined as any measure of aerobic exercise capacity (such as $\dot{V}$O_2_max or $\dot{V}$O_2_peak) or of exercise performance (e.g. a timed physical challenge). Studies were included if they evaluated exercise performance under conditions of hypoxia or changes in sea-level performance following hypoxic exposure. Case–control studies were included where cases were successful performers at >2,000 m, and where appropriate control groups (by race and by similar altitude of origin [±500 m]) were utilised. Only human studies and full primary research papers (e.g. not a conference abstract, letter to the editor, or review) were eligible for inclusion. Studies of high-altitude population genetics per se were excluded unless specifically assessing a physical performance phenotype. Research articles were excluded if they did not seek to identify genetic factors and their relationship with hypoxic exercise performance.

#### Search Strategy for Identification of Studies

The following electronic databases were searched: Ovid MEDLINE^®^ Daily Update <31 January 2014>, Ovid OLDMEDLINE^®^ <1946 to 1965>, Ovid MEDLINE^®^ In-Process and Other Non-Indexed Citations, Ovid MEDLINE^®^ <1946 to present> and Embase Classic + Embase <1947 to 31 January 2014>. Ovid MEDLINE is produced by the National Library of Medicine and indexes information from approximately 5,600 journals in the fields of biomedicine and related fields. Embase is an Elsevier database that indexes biomedical and pharmacological bibliographic records from articles published in more than 7,500 peer-reviewed journals. The search included all studies published in English and studies in any language for which the abstract was available in English. No other limits were applied.

We developed a search strategy based on the ‘patient or population, intervention, comparison, outcome’ (PICO) framework. However, the types of study we sought do not conform exactly to the PICO format, which could not therefore be fully adhered to. In particular, all populations were eligible for inclusion, making the ‘patient or population’ parameter redundant. In order to formulate a research question, each search contained one term from each of the following three parameters (intervention, comparison, and outcome) with each term linked by the Boolean operator ‘AND’. The full search strategy run in the various databases can be found in Electronic Supplementary Material Appendix S1 and Appendix S2. In brief, the key terms used in the search are listed hereafter, and these were also used to identify synonyms and related terms (which were also searched for):

- Intervention: Altitude, anoxia, or hypoxia.
- COMPARISON: Gene frequency, genotype, polymorphism, haplotype, single nucleotide polymorphism, or genetic linkage.
- Outcome: Exercise tolerance, exercise test, exercise, athletic performance, performance, mountaineering, summit, physical endurance, endurance.

#### Searching Other Resources

In addition to electronic databases, we searched bibliographies of included articles for research papers. Research papers with titles that indicated that the study was in the scope of the current review were retrieved and underwent the formal review process.

#### Data Collection and Analysis

In the first stage of the review process, two authors (PJH, AFO) independently inspected all citations from the searches (using information contained in the title, keywords and abstract) to identify candidate articles for which full texts were retrieved. If any ambiguity arose regarding the inclusion or exclusion of an article due to the absence of key information, the full-text article was obtained. In the second stage, the authors (PJH, AFO) independently applied the inclusion and exclusion criteria to the candidate full-text articles. At both stages of the process, differences regarding study inclusion were resolved through discussion.

#### Data Extraction and Management

From each source document, the lead author (PJH) extracted data including study identification information, number of participants in each group, demographic information (including the sex, age and ethnicity, race or origin of the participants), magnitude of hypoxic exposure (altitude or hypoxic exposure), polymorphism and/or gene or region being studied, gene and allele frequencies, probability values from comparisons, measure of performance, and the information required to assess the quality of each study (see Table 1). Studies were separated according to their primary study design (case–control or cohort). Odds ratios (ORs) were determined for studies with categorical outcomes variables using an additive model.

**Table 1:** Study quality scoring assessment system

Item	Criterion
1. Control group	Was the control/comparison group equal or larger in size than the case group, and was it • described in such a way that it could be replicated; • stated or inferred that the ethnicity of the control group was not different to that of the case group. If the control group from a previous study was used and referenced, the referenced study was retrieved and the control group analysed as above. No score was assigned to cohort studies for this item
2. Hardy–Weinberg equilibrium	Were the groups included in the study assessed for to determine whether they were in Hardy–Weinberg equilibrium?
3. Case group/whole group	Is the definition of the case group adequate to allow replication? For cohort studies, is the description of the whole group sufficient for replication?
4. Primer	Were the primer sequences provided or was a reference to these given?
5. Reproducibility of genotyping	Was the description of genotyping methods sufficient to allow replication or was a reference providing this information given? And, was the validity of the genotyping technique checked by performing a second assay technique, by validating the accuracy of the assay used, or was a reference to a validation study given?
6. Blinding	Were genotyping staff blinded from group allocation or phenotypic data?
7. Power calculation	Was a power calculation performed either prospectively or retrospectively?
8. Statistics	Were major findings presented with well described tests of significance including *p* values, odds ratios or confidence intervals?
9. Corrected statistics	When more than one genetic polymorphism was studied, were statistical corrections made to account for the increased risk of a false positive?^a^ One-tailed significance testing was also scored as zero. Those testing one genetic polymorphism were scored as one
10. Independent replication	Was a secondary confirmatory study performed, or does the study specifically state it is being performed to confirm results from an earlier study?

Items were scored ‘1’ for yes and ‘0’ for no

^a^If the study only investigated one genetic polymorphism, the question was scored as ‘1’

### Study Quality Analysis

The quality of each study was assessed using a scoring system (Table 1) adapted from Clark and Baudouin [4], which was based on published guidelines for conducting and reporting genetic association studies. Different aspects of the study were scored 1 if the information was present or 0 if it was not. These were then summed to give a final score that ranged from 0 to 10 for case–control studies, 0–9 for cohort studies, and 0–8 for genetic admixture studies. We modified the approach of Clark and Baudouin [4] in relation to sample size and power calculations. Studies were deemed adequate in this respect if a power calculation was reported, but we did not perform a power calculation for studies where this was not reported. The criteria used for considering the quality of the control group were also adapted. In the original system by Clark and Baudouin [4], a study that used a control group from a previously published study would need to describe the population in sufficient detail to meet the study quality criteria to score. Due to word restrictions imposed by publishers and the need to avoid repetition, we extended this. In studies that provided a reference to a control population, the referenced study was retrieved and the control group analysed according to the original criteria. We also altered the methods used to assess the reproducibility of genotyping to allocate a score both to studies that provided their own validation results and to those that provided a reference to a study using the same procedures that included validation information.

## Results

### Results of the Search

**Fig. 1:**
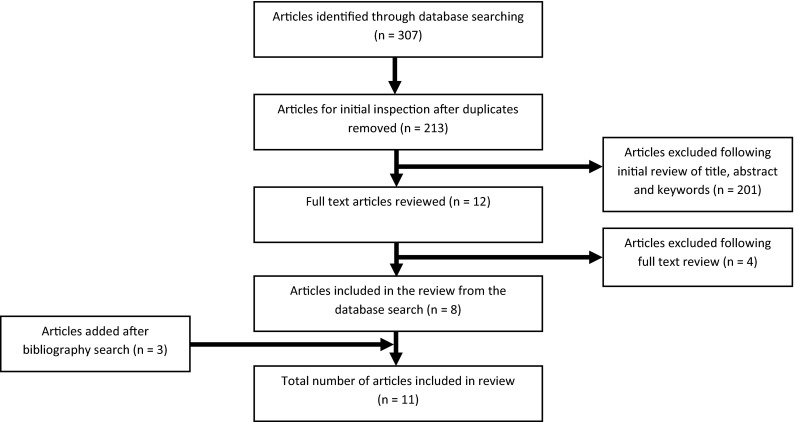
Search methodology and results

The initial search identified 307 articles (see Fig. 1). After removing duplicate articles, 213 remained for the first evaluation. Following review of the title, abstract and keywords of these records, 12 candidate articles were identified that met the predefined criteria, and full-text versions were obtained for further review. Four candidate articles did not meet the review inclusion criteria and were therefore rejected, leaving eight eligible articles for appraisal [5–12]. Examination of the full-text version of these articles and their bibliographies identified three additional eligible articles [13–15]. A total of 11 articles were included in the final analysis.

### Description of Included Studies

The 11 studies reviewed can be separated into those comparing the frequency of genotypes or alleles between two groups (case–control) (Table 2) [6, 15]; cohort studies where participants were separated according to task success or failure and where genotype or allele frequencies were compared between groups (Table 3) [8–10]; and cohort studies comparing the difference in performance measures (continuous variable) between individuals grouped by genotype or allele (Table 4) [6, 7, 11, 12]. Two studies used a different approach, and examined whether Spanish compared with Quechua genetic admixture explained differences in $\dot{V}$O_2_max at high altitude [13, 14]. According to the location of the corresponding author, studies were from the UK (*n* = 4), the USA (*n* = 3), Greece (*n* = 1), New Zealand (*n* = 1), South Africa (*n* = 1), and China (*n* = 1). The number of subjects in each study varied from 20 to 1,931, and consisted of healthy males in four studies [12–15] and healthy males and females in six studies [5, 7–11], with sex of the participants not stated in one study [6]. The maximum altitude of exposure ranged from 2,690 to 8,848 m. The exercise performance phenotype assessed was mountaineering performance in five studies [6, 8–10, 15], running performance in two studies [7, 11] and maximum oxygen consumption in four studies [5, 12–14]. In total, 13 genetic polymorphisms located within ten different genes were studied, excluding those used to estimate genetic admixture by Brutsaert and colleagues [13, 14]. The most studied gene was that encoding the angiotensin I-converting enzyme (ACE), which was studied in seven separate papers. The names of all the genes studied, excluding those used to indicate Quechua and Spanish ancestry by Brusaert and co-workers [13, 14], are listed in Table 5 along with the symbols and the chromosomal location. Results are presented according to the genetic polymorphism of interest, then by phenotype of interest (given sub-heading where necessary), and are ordered based on the maximum altitude studied.

**Table 2:** Exercise performance in atmospheric hypoxia and candidate genes; case-control studies

Gene	Polymorphism	Case			Control			*p* Value	OR	References
		N (no. of males)	Population	Frequency	*n* (no. of males)	Population	Frequency			
ACE	I/D (rs4646994)	25 (25)	Elite British mountaineers	II: 12 (0.48) ID: 11 (0.44) DD: 2 (0.08)	1,906 (1,906)	British—free from CV disease	II: (0.26) ID: (0.50) DD: (0.24)^a^	0.02	2.43	Montgomery et al. [15]
		5 (NS)	Bulgarian elite high-altitude mountaineers	II: 1 (0.20) ID: 4 (0.80) DD: 0 (0.00)	72 (NS)	Athletic Bulgarian students	II: 8 (0.11) ID: 41 (0.57) DD: 23 (0.32)	0.024–	2.29	Djarova et al. [6]
ACTN3	R577X (rs1815739)	5 (NS)	Bulgarian elite high-altitude mountaineers	RR: 1 (0.20) RX: 3 (0.60) XX:1 (0.20)	72 (NS)	Athletic Bulgarian students	RR: 30 (0.42) RX: 32 (0.44) XX: 10 (0.14)	0.032^b,c^	1.77	Djarova et al. [6]
AMPD1	G/A (rs17602729)	5 (NS)	Bulgarian elite high-altitude mountaineers	CC: 3 (0.60) CT: 1 (0.20) TT: 1 (0.20)	72 (NS)	Athletic Bulgarian students	CC: 53 (0.74) CT: 19 (0.26) TT: 0 (0.00)	0.003^b,c^	2.82	Djarova et al. [6]

*CV* cardiovascular, *NS* not stated, *OR* odds ratio

^a^Values taken from subsequent paper using the same population

^b^Statistic for allele comparisons

^c^Statistical analysis appears to be incorrect

**Table 3:** Exercise performance in atmospheric hypoxia and candidate genes: cohort studies separated by summit success

Gene	Polymorphism	Task success			Task failure			*p* Value	OR	References
		*n* (no. of males)	Population	Frequency	*n* (no. of males)	Population	Frequency			
*ACE*	I/D (rs4646994)	183 (NS—whole population: 83 % male)	Caucasians successful in summiting Mt Blanc (4,807 m)	II: 40 (0.22) ID: 93 (0.51) DD: 50 (0.27)	12 (NS)	Caucasians who failed to summit Mt Blanc (4,807 m)	II: 0 (0.00) ID: 5 (0.42) DD: 7 (0.58)	0.048	3.41	Tsianos et al. [10]
		92 (NS—whole population: 90 % male)	Caucasians and Asian mountaineers successful in summiting >8,000 m	II: 30 (0.33) ID: 41 (0.45) DD: 21 (0.23)	57 (NS)	Caucasians and Asian mountaineers unsuccessful in summiting >8,000 m	II: 3 (0.06) ID: 28 (0.60) DD: 16 (0.34)	0.003	2.15	Thompson et al. [9]
		41 (NS—whole population: 60 % male)	Successfully ascended to 5,895 m with a slow ascent profile	II: 9 (0.22) ID: 18 (0.44) DD: 14 (0.34)	41 (NS)	Unsuccessful in an attempt to summit 5,895 m with a slow ascent profile	II: 9 (0.22) ID: 22 (0.54) DD: 10 (0.24)	0.54	0.82	Kalson et al. [8]
		20 (NS—whole population: 60 % male)	Successfully ascended to 5,895 m with a fast ascent profile	II: 6 (0.30) ID: 11 (0.55) DD: 3 (0.15)	14 (NS)	Unsuccessful in an attempt to summit 5,895 m with a fast ascent profile	II: 0 (0.00) ID: 10 (0.71) DD: 4 (0.29)	0.09	2.44	Kalson et al. [8]

*ACE* angiotensin 1-converting enzyme, *I/D* insertion/deletion, *NS* not stated, *OR* odds ratio

**Table 4:** Exercise performance in atmospheric hypoxia and candidate genes: cohort studies studying a continuous trait

Gene	Polymorphism (rs number)	No. of participants (M/F)	Phenotype	Genotype frequency	Results (performance separated by genotype)	*P* Value	References
*ACE*	I/D (rs4646994)	7 Competitive runners	Sea-level time trial performance change following hypoxic training	II: 0 (0.00) ID: 3 (0.38) DD: 5 (0.62)	Sea-level time trial performance change organised by genotype was not given	Not given—NS	Hinckson et al. [7]
*ACE*	I/D (rs4646994)	142 Peruvians 68 Lowlanders (32 M) 74 Highlanders (39 M)	$\dot{V}$O_2_max at high altitude (4,438 m)	Lowlanders: II: 36 (0.53) ID: 23 (0.34) DD: 9 (0.13) Highlanders: II: 38 (0.51) ID: 31 (0.42) DD: 5 (0.07)	$\dot{V}$O_2_max (ml/kg/min)^a^ II: 35.3 (0.6) ID: 34.9 (0.8) DD: 36.0 (1.5)	0.663	Bigham et al. [5]
*AR*	AR CAG repeat polymorphism	65 Han Chinese athletes	$\dot{V}$O_2_max after hypoxic training (simulating 2,800–3,000 m altitude)	≤21 CAG repeats: 21 (0.32) >21 CAG repeats: 44 (0.68)	$\dot{V}$O_2_max (% change following training)^a^ ≤21 CAG repeats: 11.9 % >21 CAG repeats: 3.6 %	0.004	Wang et al. [12]
*ACTN3*	R577X (rs1815739)	438 Greek athletes (417 M)	Mt Olympus marathon performance	Not given	Phenotype results were not given. ND between genotypes/alleles	0.20^b,c^	Tsianos et al. [11]
*AMPD1*	G/A (rs17602729)	438 Greek athletes (417 M)	Mt Olympus marathon performance	Not given	Phenotype results were not given. ND between genotypes/alleles	0.34^b,c^	Tsianos et al. [11]
*BDKRB2*	C/T (rs1799722)	438 Greek athletes (417 M)	Mt Olympus marathon performance	Not given	Phenotype results were not given. ND between genotypes/alleles	0.17^b^	Tsianos et al. [11]
*ADRB2*	G/A (rs1042713)	438 Greek athletes (417 M)	Mt Olympus marathon performance	Not given	Phenotype results were not given. ND between genotypes/alleles	0.21^b^	Tsianos et al. [11]
*PPARGC1A*	A/G (rs8192678)	438 Greek athletes (417 M)	Mt Olympus marathon performance	Not given	Phenotype results were not given. ND between genotypes/alleles	0.71^b^	Tsianos et al. [11]
*PPARA*	G/C (rs4253778)	438 Greek athletes (417 M)	Mt Olympus marathon performance	Not given	Phenotype results were not given. ND between genotypes/alleles	0.25^b^	Tsianos et al. [11]
*PPARD*	T/C (rs6902123)	438 Greek athletes (417 M)	Mt Olympus marathon performance	Not given	Phenotype results were not given. ND between genotypes/alleles	0.24^b^	Tsianos et al. [11]
	T/C (rs1053049)	438 Greek athletes (417 M)	Mt Olympus marathon performance	Not given	Phenotype results were not given. ND between genotypes/alleles	0.20^b^	Tsianos et al. [11]
	A/G (rs2267668)	438 Greek athletes (417 M)	Mt Olympus marathon performance	Not given	Phenotype results were not given. ND between genotypes/alleles	0.71^b^	Tsianos et al. [11]
*APOE E4*	(rs7412)	438 Greek athletes (417 M)	Mt Olympus marathon performance	Not given	Phenotype results were not given. ND between genotypes/alleles	0.12^b^	Tsianos et al. [11]
	(rs429358)	438 Greek athletes (417 M)	Mt Olympus marathon performance	Not given	Phenotype results were not given. ND between genotypes/alleles	>0.05^b^	Tsianos et al. [11]

See Table 5 for gene names

*AR* androgen receptor, *F* female, *I/D* insertion/deletion, *M* male, *ND* no difference, *NS* not significantly different, $\dot{V}$ *O*_*2*_ *max* maximum oxygen consumption

^a^Data are presented as mean (standard error)

^b^Probability value only for male athletes as values not given for the whole group

^c^Significant differences were found between genotypes for other subgroup analysis

**Table 5:** Symbols, names, and chromosomal location of all genes identified

Gene	Name	Location
*ACE*	Angiotensin 1-converting enzyme	17q23
*ACTN3*	Actinin, alpha 3	11q13–q14
*ADRB2*	Adrenergic, beta 2, receptor	5q31–q32
*AMPD1*	Adenosine monophosphate deaminase 1	1p13
*APOE*	Apolipoprotein E	19q13.2
*AR*	Androgen receptor	Xq11–12
*BDKRB2*	Bradykinin receptor B2	14q32.1–q32.2
*PPARA*	Peroxisome proliferative activated receptor, alpha	22q13.31
*PPARD*	Peroxisome proliferative activated receptor, delta	6p21.2–p21.1
*PPARGC1A*	Peroxisome proliferative activated receptor, gamma, coactivator 1, alpha	4p15.1

### Angiotensin-Converting Enzyme (ACE) Insertion/Deletion (I/D) Polymorphism (rs4646994)

Seven studies investigated the association of the ACE I/D (rs4646994) polymorphism with exercise performance at altitude. Two were case–controlled studies that compared genotype or allele frequencies between high-altitude mountaineers and a control population (Table 2). Five cohort studies were also conducted; in three of these, study participants were separated according to task success or failure, and genotype and/or allele frequencies were compared between groups (Table 3), whilst two studies compared performance phenotype (as a continuous variable [e.g. time and $\dot{V}$O_2_max]) between those of different genotype (Table 4).

#### Mountaineering Performance

In a prospective study, Tsianos et al. [10] reported the ACE I-allele to be associated with successful ascent of Mont Blanc (4,807 m). ACE genotype distribution varied between Caucasian climbers who successfully summited Mt Blanc (*n* = 183) and those who tried and failed (*n* = 12) (*p* = 0.048) (OR 3.41), the I allele being more prevalent in those who summited than in those who did not (0.47 vs 0.21, *p* = 0.01) (Table 3). The proportion of those of each genotype who successfully summited was 87.7, 94.9 and 100 % for the DD, ID, and II genotypes, respectively.

Kalson et al. [8] prospectively studied Caucasian trekkers attempting to climb Mt Kilimanjaro (5,895 m), participants being separated by ascent rate (4 days [‘direct’] vs. 5 days [‘slow’]). Genotype distribution did not differ by success rate in the ‘slow’ group (II: 9 [0.22], ID: 18 [0.44], DD: 14 [0.34] successful versus II: 9 [0.22], ID: 22 [0.54], DD: 10 [0.24] unsuccessful, *p* = 0.54; I allele frequency 0.44 vs. 0.49, respectively; OR 0.82). In ‘fast ascent’ subjects, ACE genotype distribution was II: 6 (0.30) vs. ID: 11 (0.55) vs. DD: 3 (0.15) in those who were successful and II: 0 (0.00) vs. ID: 10 (0.71) vs. DD: 4 (0.29) in those who failed (*p* = 0.09). I-allele frequency for those who were successful versus those who failed was 0.58 vs. 0.36 (OR 2.44) (Table 3).

Montgomery et al. [15] studied ACE genotype in 25 elite British high-altitude mountaineers who had frequently ascended beyond 7,000 m without supplementary oxygen. When compared with 1,906 British controls (free from clinical cardiovascular disease), the I allele was found to be over-represented in cases (*p* = 0.003), as was the ACE II genotype (proportion 0.48 vs. 0.23 in cases vs. controls, respectively) (OR 2.43) (Table 2). The DD genotype was not present in any individual who had previously ascended beyond 8,000 m.

Djarova et al. [6] also reported I allele prevalence to be greater in five elite Bulgarian mountaineers (all climbed above 7,500 m without supplementary oxygen) than in 72 Bulgarian student controls (0.60 vs. 0.41, respectively; *p* = 0.002) (OR 2.29). ACE genotype distribution differed, largely due to an excess of ID genotype in the elite group (*p* = 0.024) (Table 2). The small case population (*n* = 5) limits confidence in this finding. Further, we were unable to replicate their statistical analysis: using the ACE allele frequencies and population numbers stated in the paper, and applying Fisher’s exact test (as the authors did) in a commonly used statistical package (SPSS 21 [IBM, Armonk, NY, USA]), we were unable to demonstrate a statistically significant difference in ACE allele frequencies between the two groups (*p* = 0.203). The same held true for the two other polymorphisms they studied, which were the alpha actinin-3 ACTN3 R577X (rs1815739) and the adenosine monophosphate deaminase (AMPD1) C34T polymorphisms (rs17602729).

Thompson et al. [9] extended the observations of Tsianos et al. [10], Kalson et al. [8] and Montgomery et al. [15] to individuals attempting to climb beyond 8,000 m, also noting the maximum altitude attained by each individual and the reason for failure. Once again, in a population of Caucasian and Asian mountaineers, ACE genotype distribution differed between successful summiteers (*n* = 92) and those who failed (*n* = 57) (OR 2.15) (Table 3) (*p* = 0.003). Both II genotype and the I allele were more prevalent in the successful group (II genotype 0.33 vs. 0.06 [*p* = 0.003]; I allele, 0.55 vs. 0.36 for success vs failure, respectively). Maximum altitude attained (as a continuous variable) was also related to ACE I allele, being 8,079 vs. 8,107 and 8,559 m for those of DD, ID and II genotype, respectively (allele comparison, *p* = 0.0023).

#### Maximum Oxygen Uptake ($\dot{V}$O_2_max) at Altitude

Bigham et al. [5] reported the relationship of ACE genotype with $\dot{V}$O_2_max at 4,338 m in Peruvian high-altitude native residents (*n* = 74) and in Peruvian low-altitude residents whose family originate from high altitude (*n* = 68). $\dot{V}$O_2_max did not differ by genotype, even when other cofactors, such as altitude of residence, were accounted for (Table 4).

#### Sea-Level Running Performance

Hinckson et al. [7] studied whether improvement in sea-level exercise performance following hypoxic exposure (2,500–3,500 m) was related to ACE genotype (Table 4). This study was primarily designed to test the efficacy of hypoxic training for improving running performance. It included a study group whose ten participants were exposed to 4 weeks of hypoxia for >10 h per day, and ten controls who had no hypoxic exposure. In eight participants (ethnicity not disclosed) in the study group, the relationship between improvements in running performance following hypoxic training with ACE I/D genotype was sought (Table 3). However, neither numerical results by genotype nor statistical analysis are reported—although a figure suggests a lack of genotype association.

### ACTN3 R577X (rs1815739)

Two studies tested whether an association was present between the ACTN3 R577X polymorphism and high-altitude exercise performance. Tsianos et al. [11] included the ACTN3 RX polymorphism as one of 11 polymorphisms (in eight genes) for which an association was sought with performance in the Mt Olympus Marathon (maximum altitude of 2,690 m). In all, no such association was identified in 438 participants over 2 years of racing. The distribution of the ACTN genotypes in the small elite high-altitude mountaineer cohort (*n* = 5) tested by Djarova et al. [6] was RR 1 (0.20), RX 3 (0.60), and XX 1 (0.20) compared with RR 30 (0.42), RX 32 (0.44) and XX 10 (0.14) in the control group (*n* = 72). The allele frequencies between the two groups were different (*p* = 0.032), with a relative excess of the X allele in the mountaineers (OR 1.77) (Table 2). However, as discussed in the previous section, the statistical approach of Djarova et al. [6] was of questionable validity. Using the allele frequencies and subject numbers from the paper [6], we found no difference in the ACTN RX allele distributions between the two populations (*p* = 0.501).

### AMPD1 C34T (rs17602729)

In the study by Tsianos et al. [11] (see previous section), the AMPD C34T polymorphism was not associated with Mt Olympus Marathon performance for the whole group studied, but subgroup analysis revealed a modest association between the T allele (referred to as the ‘A allele’ in Tsianos et al. [11]) and better performance in athletes whose preferred sport was running (event completion time, *p* = 0.021; best ever time, *p* = 0.03). Djarova et al. [6] found that the frequency of the T allele was higher in mountaineers than in controls (*p* = 0.032; OR 2.82) (Table 2). However, as stated in the previous two results sections, the statistical analysis in this study is incorrect; using the reported allele frequencies in a Fisher’s test, we found no difference in the distribution of the allele of the AMPD1 C34T polymorphism between the elite mountaineers and controls (*p* = 0.155).

### Other Polymorphisms

Ten other polymorphisms were studied. Of nine studied by Tsianos et al. [11] (listed in Table 4), the beta2-adrenergic receptor (ADRB2) Gly16Arg single nucleotide polymorphism (SNP) (rs1042713) was associated with performance in the Mt Olympus Marathon. In the study by Tsianos et al. [11], the Arg allele was associated with a shorter time to complete the race amongst people who chose running as their preferred exercise mode (*p* = 0.015), and with best ever Mt Olympus Marathon time for those who had completed the event multiple times (*p* = 0.003). Meanwhile, Wang et al. [12] studied the association between the androgen receptor (AR) CAG repeat polymorphism and $\dot{V}$O_2_max changes in 65 unrelated Han Chinese college athletes following a 30-day bout of intermittent hypoxic exposure, consisting of 10 h a day of sleeping at 14.8–14.3 % O_2_ (simulating 2,800–3,000 m altitude), 30-minute exercise bouts at 15.4 % O_2_ (simulating 2,500 m altitude) three times per week, and additional sea-level training. They found that individuals with the lowest number of CAG repeat units (≤21) had a greater increase in $\dot{V}$O_2_max following hypoxic training than those with a higher number of repeats (>21) (∆$\dot{V}$O_2_max; ≤21 CAG repeats 11.9 % increase, >21 CAG repeats 3.6 % increase; *p* = 0.004).

### High-Altitude Population Ancestry

Two papers from the same group measured a number of physiological variables, including $\dot{V}$O_2_max, at altitude in Peruvian males of mixed Quechua and Spanish origins [13, 14]. Both sought to determine whether individuals with stronger Quechua (historical long-term high-altitude resident) origins had favourable physiological responses to altitude. We considered excluding these two papers as, strictly speaking, they do not meet one of the inclusion criteria: to ‘seek to identify genetic factors.’ However, we did include them given that they studied exercise performance at altitude and separated individuals based on their genetic profile. The first, published in 2004, comprised two groups from Peru; a group resident in Lima (sea level) and a group resident in Cerro de Pasco (4,388 m) [13]. No association was found between $\dot{V}$O_2_max values at an altitude of 4,388 m, and the degree of Quechua, Spanish and African ancestry, as indicated by 22 genetic markers. The second study [14] used the same lowland population and the same genetic profiling as the 2004 study [13] (confirmed with primary author of the manuscripts via email correspondence) and analysed the influence of genetic admixture on the magnitude of the $\dot{V}$O_2_max decrement from sea level to high altitude (data not presented in the same author’s 2004 paper). After controlling for other covariates, genetic admixture explained a significant proportion of the variation (*β* −0.205) in the decrement in $\dot{V}$O_2_max from sea level to high altitude (*p* = 0.041), with those with a higher Quechua (and lower Spanish) genetic ancestry maintaining sea-level $\dot{V}$O_2_max values to a greater degree.

### Study Quality

As the method of assessing study quality is not validated and may only provide a guide to the quality of reporting of the research, we have not commented on each study individually. Instead, we aim to identify trends in the available literature where the conduct and/or reporting of research could be improved. The median score achieved in the study quality analysis was 6 out of 10 for case–control studies, 8 out of 10 for cohort studies with a discrete outcome, 6 out of 9 for cohort studies with a continuous outcome, and 4.5 out of 8 for genetic admixture studies (Table 6). The study quality score ranged considerably between studies, for example in cohort studies with a continuous outcome the total score ranged from 2 to 7 out of 9.

**Table 6:** Study quality results

Study ID	Control group	HW equilibrium	Case group/whole group	Primer	Reproducibility of genotyping	Blinding	Power calculation	Statistics	Corrected statistics	Independent replication	Total
Case–control											
Montgomery et al. [15]	1	1	1	1	1	0	0	1	1	0	7/10
Djarova et al. [6]	0	1	1	1	0	0	1	1	0	0	5/10
Cohort: discrete outcome											
Tsianos et al. [10]	0	1	1	1	1	0	0	1	1	1	7/10
Thompson et al. [9]	0	1	1	1	1	1	0	1	1	1	8/10
Kalson et al. [8]	0	1	1	1	0	1	1	1	1	1	8/10
Cohort: continuous outcome											
Hinckson et al. [7]	–	0	1	0	0	0	0	0	1	0	2/9
Bigham et al. [5]	–	1	1	1	1	0	0	1	1	0	6/9
Tsianos et al. [11]	–	1	1	0	1	0	1	1	1	1	7/9
Wang et al. [12]	–	1^a^	1	1	1	0	0	1	1	0	6/9
Genetic admixture											
Brutsaert et al. [14]	–	–	1	0	1	0	0	1	1	0	4/8
Brutsaert et al. [13]	–	–	1	0	1	0	1	1	1	0	5/8

*HW* Hardy–Weinburg

^a^Polymorphism located on the X chromosome and the populations were male, so genotype distribution would not conform with Hardy-Weinburg equilibrium, this section was scored as 1

In the case–control studies, the descriptions of the case groups were adequate, with both the studies given a score of 1 [6, 15]. However, only one of the control groups was deemed adequate [15], with the other study failing to provide adequate information about whether the level of relatedness of participants was controlled [6]. The majority of studies in which testing for Hardy–Weinberg (HW) equilibrium was appropriate did so (seven of eight). The one study that did not test for HW equilibrium had very small subject numbers (*n* = 8) and so it would have provided very little information [7]. In the other three studies, two investigated whether genetic admixture was associated with performance. This approach utilised polymorphisms that differed according to ancestry, meaning that testing for HW equilibrium would be uninformative [13, 14]. In the other study, the polymorphism was located on the X chromosome, and the populations were male, so genotype distribution would not conform with HW equilibrium, evidence of which was thus not sought [12].

Three study quality criteria relate to genotyping methods and reporting: providing the sequence of primers, demonstrating the reproducibility of the genotyping methods, and adequately blinding the genotyping staff from the phenotype details. Most studies (7 of 11) provided the details of the primer sequence (or a reference to them), one stated that this would be made available on request [11], two gave reference to a website that could be used to identify them [13, 14], and one failed to provide any information [7]. Eight studies stated that they had validated their genotyping methods or cited a reference for a validation study, while only two studies stated that their genotyping staff were blinded from the phenotype data.

The statistical merit of each paper was analysed in three of the study quality criteria. These analysed whether appropriate statistical tests were performed, whether adjustments were made to account for multiple comparisons in studies analysing more than one polymorphism, and whether power calculations were made. Ten of the 11 studies used appropriate formal methods of comparison to establish whether difference were present between groups. Only one study did not use a formal test of significance, and this was probably justified because the small number of participants would have precluded any meaningful results being obtained [7]. Only one study analysed several polymorphisms and failed to make adjustments for multiple comparisons [6]. Four of the 11 studies conducted sample size calculations [6, 8, 11, 13], with one study performing the calculation prior to conducting the study [11].

No article included in the review provided both a study identifying a potential polymorphism and a study validating the original finding. Following the original paper analysing the association between the ACE I/D polymorphism and mountaineering performance by Montgomery et al. [15], several other papers analysed the association between this polymorphism and highly related phenotypes [6, 8–10]. Furthermore, one study replicated polymorphisms related to endurance performance at sea level using an endurance race at altitude as the phenotype [11].

## Discussion

### Main Findings

This review identified four genetic polymorphisms associated with hypoxic exercise performance: AMPD1 C34T, ADRB2 GA, AR CAG repeat, and the ACE I/D polymorphisms. Support for the association between the ACE I allele and high-altitude performance was the strongest, with three studies identifying a positive relationship between the I allele and improved performance. Conversely, the AMPD1 34T allele and the ADRB2 16Arg allele were associated with better performance in atmospheric hypoxia in one study, while those with the lowest number of CAG repeat units in the AR had better $\dot{V}$O_2_max gains following hypoxic training in one study. This review identified that the number of articles and the total number of polymorphisms studied within this area are few; only 11 articles were identified and only 13 polymorphisms within ten different genes studied. In addition, the conduct and/or reporting of the studies was not optimal, with many failing to select and describe the populations adequately, several not conducting or describing their genotyping methods sufficiently and many conducted without calculating the correct sample size required to adequately power the study.

### Study Number and Design

The small number of articles identified in the current review and the limited number of polymorphisms studied in total highlights that the influence of genetic factors on exercise performance in hypoxia has not been studied in depth. This is in contrast to the hundreds of articles that have studied the influence of genetics on other phenotypes such as exercise performance at sea level [16]. The majority of studies have taken the candidate gene approach (*n* = 9), whilst two studied whether genetic admixture influenced exercise performance [13, 14]. The limited range of SNPs studied is likely due to a bias towards studying genes associated with exercise and/or altitude phenotypes in previous publications and to the reliance on the candidate gene approach. The absence of genome-wide association studies is notable, as it would allow hypothesis-free investigation and help identify novel candidate genes for which the biological pathway is not known. This approach is commonplace in many other fields of research and has been used successfully to identify polymorphisms associated with $\dot{V}$O_2_max gains at sea level [17], obesity [18] and blood pressure [19]. The candidate gene approach is justified, as it has greater power to detect difference in genotype frequency between groups, and is suited to studies using modest population sizes [20]. Despite most studies using the candidate gene method, study designs differed greatly with regards to the population, hypoxic exposure, and the performance phenotype. The small number of studies, in addition to the variety of study designs, makes comparison between studies difficult, and limits the ability to assess the reliability of results. The variety of study designs, polymorphisms and outcomes studied, and the small number of studies, precluded meaningful formal assessment of bias, such as a funnel plot [21]. That said, in spite of small sample sizes, seven of the nine candidate gene studies identified an association between a polymorphism and the performance phenotype with effect sizes considered very large for genetic association studies [22]. These factors may point towards some form of bias within the literature base, although this cannot be confirmed at this time.

### Genetic Polymorphisms Associated with Exercise Performance during Hypoxic Exposure

This review identified four polymorphisms associated with exercise performance in a hypoxic environment, and found nine polymorphisms to have no relationship (Tables 3, 4, 5). Furthermore, in a study of Peruvian natives, a genetic profile with a greater Quechua (as opposed to Spanish) origin was associated with greater preservation of $\dot{V}$O_2_max at altitude [14].

### ACE I/D Polymorphism (rs4646994)

The ACE I/D polymorphism had the most supporting literature, with three studies observing an association of the I allele (lower ACE activity) with mountaineering performance [9, 10, 15]. ACE is an important component of the endocrine renin-angiotensin system (RAS), converting angiotensin I to angiotensin II, which exerts pressor effects through arteriolar vasoconstriction and aldosterone-mediated salt and water retention [23]. However, cellular and tissue RAS are now known to exist in multiple tissues [23], where they have diverse roles relating to the regulation of cell growth and survival, metabolism and inflammation [24]. The presence (insertion, I-allele) rather than absence (deletion, D-allele) of a 287 bp fragment in the ACE gene (rs4646994) is associated with lower circulating [25] and tissue [26, 27] ACE activity. Whilst the mechanism underpinning the association between the ACE I/D polymorphism and mountaineering performance has yet to be fully established, the I allele has been associated with enhanced endurance performance at sea level [28], greater hypoxic ventilatory drive [29], enhanced arterial oxygenation at altitude [30], and enhanced training-related gains in metabolic efficiency [31].

It should be noted that three studies found no association between the ACE ID polymorphism and their hypoxic exercise performance phenotype of interest [5, 7, 8] and one presented incorrect results [6]. The others found no ACE genotype association with $\dot{V}$O_2_max at 4,338 m in Peruvian natives [5] or change in endurance running performance following hypoxic training [7]. However, this later study had limited power to detect any difference in genotype distribution due to a very small sample size. These results are important in their own right, but it is entirely plausible that an association exists between the ACE polymorphism and mountaineering performance that is mediated by a mechanism unrelated to $\dot{V}$O_2_max. The lack of association between the polymorphism and mountaineering performance in the study by Kalson et al. [8] is more difficult to explain. However, despite the altitude exposure and performance phenotype being similar in all four studies, there are still subtle differences in ascent rate, maximum altitude of exposure and total time at high altitude, which may explain the discrepancy. Furthermore, Kalson et al. [8] acknowledged that they had insufficient sample size to detect a difference between successful and unsuccessful climbers and were therefore at risk of a type 2 error. The lack of power due to small sample size is a limitation of many of the reviewed studies and will be discussed further in the ‘Study Quality’ section.

### ACTN3 R577X (rs1815739)

The *ACTN3* gene encodes the protein alpha actinin-3, an actin-binding protein expressed predominantly in type II (fast) muscle fibres, which has structural, cell signalling and metabolic roles [32, 33]. The *ACTN3* gene contains a nonsense polymorphism that results in the substitution of an arginine (R) with a stop codon (X) at amino acid 577 on the alpha actinin-3 protein (rs1815739) [34]. The premature termination of the alpha actinin-3 protein associated with homozygosity for the R577X null allele results in complete alpha actinin-3 deficiency, and occurs in approximately 16 % of the world population [34]. Whilst this deficiency does not cause muscle disease, possibly due to compensation by the closely related isoform alpha actinin-2, it does appear to influence sporting performance [32]. The frequency of the ACTN3 alleles was reported to differ between a ‘normal’ population, an elite sprint and power athletic population, and an elite endurance athletic population (*p* < 0.001), with elite power athletes having a lower frequency of the XX genotype (normal 18 %; elite power 6 %) and elite endurance athletes having a higher frequency of XX genotype (normal 0.16; elite endurance 0.24) [35]. The two studies identified in our review do not support an association between the ACTN3 R577X polymorphism and exercise performance at high altitude [6, 11]. However, given the limited performance phenotypes investigated and the flawed statistical analysis in the study by Djarova et al. [6], an association cannot be excluded.

### AMPD1 C34T (rs17602729)

AMPD catalyses the conversion of adenosine monophosphate (AMP) to inosine monophosphate (IMP) and ammonia which, in turn, moves the myokinase equilibrium reaction (2 ADP ⇌ ATP + AMP) towards ATP re-synthesis [36]. The *AMPD1* gene contains a genetic variation (C34T) that results in the replacement of a glutamine amino acid with a stop codon and has an allele frequency of 13.7 % in a healthy Caucasian population [37]. Norman et al. [37] also showed that AMPD activity in skeletal muscle of AMPD1 TT homozygotes was <1 % that of CC homozygotes, and that heterozygotes had intermediate activity levels. At sea level, the AMPD1 TT homozygotes have been shown to have ~14 % lower peak power output than their CT/CC counterparts (*p* < 0.05) [38], and the frequency of the T allele is lower in elite endurance athletes than in controls (0.043 vs. 0.085; *p* < 0.05) [39]. Tsianos et al. [11] also found an association between the AMPD T allele and Mt Olympus Marathon performance in athletes whose preferred endurance sport was running. It is thus unclear whether AMPD1 genotype is associated with endurance performance per se or with hypoxic endurance performance. No data are available to answer this question. The results must be viewed with some caution, as they were obtained in a sub-population of the whole group studied and are yet to be verified in other high-altitude exercise phenotypes (we do not consider the results of Djarova et al. [6] valid).

### Other Polymorphisms

The adrenergic receptors are G protein-coupled receptors that are expressed in a wide range of tissues and cause a variety of physiological responses in response to binding with circulating catecholamines [40]. The ADRB2 subgroup has a primary function to facilitate a sympathetic nervous system response, altering respiratory, cardiac and vascular function [41]. In a meta-analysis, the pooled frequency of the ADRB2 minor (16Arg) allele was 0.420 in Caucasians, 0.492 among populations with an African heritage (African-American, African and Afro-Caribbean populations) and 0.562 among Oriental populations [42]. The ADRB2 polymorphism alters physiological function; compared with their ADRB2 Gly/Gly counterparts, 16Arg homozygotes have lower receptor density on isolated lymphocytes [43], lower resting stroke volume and cardiac output [43, 44], and lower sub-maximal exercising stroke volume and mean arterial pressure [44]. Furthermore, the Arg/Arg genotype was more prevalent in an elite male endurance athlete population than in a control population consisting of geographically matched males (0.17 vs. 0.09, *p* = 0.03).

This review identified one study in which the ADRB2 Arg allele was associated with better endurance exercise performance at altitude. Tsianos et al. [11] found the Arg allele was associated with better performance in the Mt Olympus Marathon amongst people whose preferred mode of exercise was running, and with best ever Mt Olympus Marathon time amongst those who had completed the event several times. As described in the previous section regarding similar results for the AMPD1 C34T SNP, these results should be viewed with caution given that these associations were established for particular subgroups and were only sought and identified in one study. However (and again as described above), the results are aligned with those obtained at sea level, which increases the confidence in the validity of such findings.

Androgens, such as testosterone, act by binding to the AR, and their effects on anthropometric variables and physical performance (including increasing fat free mass, muscle strength and size) are well described [45]. In skeletal muscle cells, the AR CAG repeat polymorphism alters the receptor’s transcriptional activity, with higher repeat lengths causing greater activity, as well as altering markers of cell growth and development [46]. Wang et al. [12] identified an association between those with a lower number of CAG repeat units (≤21) and a greater increase in $\dot{V}$O_2_max following hypoxic training. However, a normoxic control population was not employed and therefore the increases in $\dot{V}$O_2_max, and the association with the AR polymorphism, may simply reflect a training effect. Further studies designed to distinguish the effect of training from that of hypoxia are required.

### High-Altitude Population Ancestry

The two related studies by Brutsaert and colleagues [13, 14] found no association between the degree of Quechua, Spanish and African ancestry in a population of Peruvian males, as indicated by 22 genetic markers and $\dot{V}$O_2_max values at an altitude of 4,388 m. However, they did find that the magnitude of the decrease in $\dot{V}$O_2_max from sea level to high altitude was lower in those with a greater degree of Quechua ancestry. The specific mechanisms to explain this exercise performance phenotype are not readily forthcoming as the data in this field are sparse; however, a genetically mediated reduction in arterial oxygen saturation (SaO_2_) during maximal exercise appears to have a role [14]. This finding adds to a growing body of literature suggesting that the Quechua have altered anthropological and physiological responses to hypoxia as a result of genetic selection [47].

### Study Quality

Analysis of study quality highlights a need for better conduct and/or reporting of genetic association studies in this field. The selection of populations, genotyping practices, and statistical procedures were inadequate in many of the studies (Table 6), and only three studies attempted to replicate prior findings using similar phenotypes [8–10]. Some studies did not ensure that ethnicity of populations was accounted for in the analysis process, country of origin often being an inadequate descriptor. Selecting unrelated populations of the same ethnicity is critical to avoid population stratification, which can confound results and lead to spurious association being identified [48]. However, such problems are hard to overcome in ‘field’ research. Poor genotyping practices may also lead to erroneous results and can be caused by many factors including collecting low quality and/or quantity of DNA, machine error or failure, human error, poor storage conditions, and issues with the array [49]. Despite this, only eight studies stated that they had checked the validity of their genotyping procedure or cited a reference for a validation study [5, 9–12, 14, 15], and only two blinded genotyping staff from phenotypic data [8, 9]. Meanwhile, seven studies provided sufficient primer information to allow replication. Seven of the 11 studies failed to conduct sample size calculations, and only one study performed the calculation prior to conducting the study. The majority of studies included in the review seem underpowered to test their hypothesis effectively as the associations between SNPs and complex human phenotypes, such as exercise performance, are generally small [22]. The studies in this review typically had a sample size of under 100, whereas it has been suggested that to adequately power genetic association studies investigating complex multifactorial traits, studies should employ sample sizes well in excess of this number [22]. Finally, replication of initial findings is of paramount importance because a first study often overestimates the size of the effect when compared with subsequent studies, and in many cases the subsequent studies fail to support the initial findings [50]. The studies we reviewed failed to validate initial findings by testing the same genetic polymorphism on a similar population and phenotype. However, the association between the ACE polymorphism and mountaineering performance identified by Montgomery et al. [15] was evaluated in similar populations in three subsequent studies, albeit using different study designs [8–10].

### Strengths and Limitations of the Review

The strengths of this review are that it used systematic search and analysis methodologies to identify studies that have investigated the association between genetic factors and exercise performance in atmospheric hypoxia. This allowed us to identify all studies in the area and avoided selection bias. Identifying these polymorphisms will guide future research, identifying targets for further study and helping to avoid the repetition of work. The review also analysed the conduct and/or reporting of the studies, giving an indication of study quality and highlighting where improvements in study design can be made.

Limitations of this study include the method of study selection. It may have been helpful to exclude studies based on study design and/or quality to provide a clearer picture of which polymorphisms and molecular systems are important in hypoxic exercise performance. However, the lack of a robust validated method of analysing study design and quality in genetic association studies made this difficult. Consequently, we adapted Clark and Baudouin’s [4] method of analysing study quality. This method has not been validated and thus it is unclear whether it indicates greater study validity, or the likelihood of replication. However, it does follow previous studies and articles that give recommendations for how genetic association studies should be conducted and presented [51, 52].

## Conclusions

This review shows that the association between genetic polymorphisms and high-altitude exercise performance has not been studied in depth. We identified only 11 articles that studied a total of just 13 genetic polymorphisms. Four polymorphisms were associated with hypoxic exercise performance: AMPD1 C34T, ADRB2 GA, AR CAG repeat, and the ACE I/D polymorphisms. The strongest association appears to be between the ACE I allele and enhanced high-altitude mountaineering performance, with three studies identifying a relationship. The study quality analysis highlighted deficiencies in the conduct and reporting of the genetic association studies. The selection and description of populations, genotyping methods, and statistical analysis procedures were inadequate in many of the studies. In particular, many studies failed to calculate the correct sample size required to adequately power their study and subsequently most appear underpowered to test their hypothesis effectively. To attain greater understanding of the role that genetic factors have on high-altitude exercise performance, further studies need to be performed. Future studies should endeavour to use more robust study design (e.g. appropriate sample size, replicable phenotypes, robust methods of selecting study and control populations) and ensure they provide adequate information to permit subsequent replication or meta-analysis. Furthermore, genome-wide studies should be considered in future in order to help identify novel biological pathways of hypoxic adaptation.

## Electronic supplementary material

Below is the link to the electronic supplementary material.

Supplementary material 1 (DOCX 12 kb)

Supplementary material 2 (DOCX 12 kb)
