# Supplementary material for: Genetic Factors Associated with Exercise Performance in Atmospheric Hypoxia
Source: Sports Med. 2015 Feb 15;45(5):745–61. doi: 10.1007/s40279-015-0309-8 (PMC4544548; doi:10.1007/s40279-015-0309-8)
Supplement: Supplementary file 1 — Supplementary material 1 (DOCX 12 kb) [file 40279_2015_309_MOESM1_ESM.docx]

Electronic Supplementary Material Appendix S1: Ovid Medline search strategy

Database: Ovid MEDLINE(R) Daily Update <January 31, 2014>, Ovid OLDMEDLINE(R) <1946 to 1965>, Ovid MEDLINE(R) In-Process & Other Non-Indexed Citations and Ovid MEDLINE(R) <1946 to Present>

Search Strategy:

--------------------------------------------------------------------------------

1 exp Altitude/ (13102)

2 altitude.mp. [mp=title, abstract, original title, name of substance word, subject heading word, keyword heading word, protocol supplementary concept word, rare disease supplementary concept word, unique identifier] (21000)

3 exp Anoxia/ (53666)

4 hypoxia.mp. [mp=title, abstract, original title, name of substance word, subject heading word, keyword heading word, protocol supplementary concept word, rare disease supplementary concept word, unique identifier] (84808)

5 1 or 2 or 3 or 4 (124044)

6 exp Gene Frequency/ (57788)

7 (gene adj1 frequency).mp. [mp=title, abstract, original title, name of substance word, subject heading word, keyword heading word, protocol supplementary concept word, rare disease supplementary concept word, unique identifier] (55312)

8 exp Genotype/ (279601)

9 genotype.mp. [mp=title, abstract, original title, name of substance word, subject heading word, keyword heading word, protocol supplementary concept word, rare disease supplementary concept word, unique identifier] (198591)

10 exp Polymorphism, Genetic/ (188145)

11 polymorphism.mp. [mp=title, abstract, original title, name of substance word, subject heading word, keyword heading word, protocol supplementary concept word, rare disease supplementary concept word, unique identifier] (221924)

12 exp Haplotypes/ (33762)

13 haplotype.mp. [mp=title, abstract, original title, name of substance word, subject heading word, keyword heading word, protocol supplementary concept word, rare disease supplementary concept word, unique identifier] (32204)

14 (single adj1 nucleotide adj1 polymorphism).mp. [mp=title, abstract, original title, name of substance word, subject heading word, keyword heading word, protocol supplementary concept word, rare disease supplementary concept word, unique identifier] (63796)

15 exp Genetic Linkage/ (51478)

16 (genetic adj1 linkage).mp. [mp=title, abstract, original title, name of substance word, subject heading word, keyword heading word, protocol supplementary concept word, rare disease supplementary concept word, unique identifier] (39374)

17 6 or 7 or 8 or 9 or 10 or 11 or 12 or 13 or 14 or 15 or 16 (482117)

18 exp Exercise Tolerance/ (7775)

19 exp Exercise Test/ (49008)

20 exercise.mp. [mp=title, abstract, original title, name of substance word, subject heading word, keyword heading word, protocol supplementary concept word, rare disease supplementary concept word, unique identifier] (226265)

21 exp Athletic Performance/ (37013)

22 performance.mp. [mp=title, abstract, original title, name of substance word, subject heading word, keyword heading word, protocol supplementary concept word, rare disease supplementary concept word, unique identifier] (572610)

23 exp Mountaineering/ (2191)

24 mountaineer*.mp. [mp=title, abstract, original title, name of substance word, subject heading word, keyword heading word, protocol supplementary concept word, rare disease supplementary concept word, unique identifier] (2459)

25 summit.mp. [mp=title, abstract, original title, name of substance word, subject heading word, keyword heading word, protocol supplementary concept word, rare disease supplementary concept word, unique identifier] (2589)

26 exp Physical Endurance/ (23651)

27 endurance.mp. [mp=title, abstract, original title, name of substance word, subject heading word, keyword heading word, protocol supplementary concept word, rare disease supplementary concept word, unique identifier] (25489)

28 18 or 19 or 20 or 21 or 22 or 23 or 24 or 25 or 26 or 27 (796079)

29 5 and 17 and 28 (123)

30 exp Altitude/ (13102)

31 altitude.mp. [mp=title, abstract, original title, name of substance word, subject heading word, keyword heading word, protocol supplementary concept word, rare disease supplementary concept word, unique identifier] (21000)

32 exp Anoxia/ (53666)

33 hypoxia.mp. [mp=title, abstract, original title, name of substance word, subject heading word, keyword heading word, protocol supplementary concept word, rare disease supplementary concept word, unique identifier] (84808)

34 30 or 31 or 32 or 33 (124044)

35 exp Gene Frequency/ (57788)

36 (gene adj1 frequency).mp. [mp=title, abstract, original title, name of substance word, subject heading word, keyword heading word, protocol supplementary concept word, rare disease supplementary concept word, unique identifier] (55312)

37 exp Genotype/ (279601)

38 genotype.mp. [mp=title, abstract, original title, name of substance word, subject heading word, keyword heading word, protocol supplementary concept word, rare disease supplementary concept word, unique identifier] (198591)

39 exp Polymorphism, Genetic/ (188145)

40 polymorphism.mp. [mp=title, abstract, original title, name of substance word, subject heading word, keyword heading word, protocol supplementary concept word, rare disease supplementary concept word, unique identifier] (221924)

41 exp Haplotypes/ (33762)

42 haplotype.mp. [mp=title, abstract, original title, name of substance word, subject heading word, keyword heading word, protocol supplementary concept word, rare disease supplementary concept word, unique identifier] (32204)

43 (single adj1 nucleotide adj1 polymorphism).mp. [mp=title, abstract, original title, name of substance word, subject heading word, keyword heading word, protocol supplementary concept word, rare disease supplementary concept word, unique identifier] (63796)

44 exp Genetic Linkage/ (51478)

45 (genetic adj1 linkage).mp. [mp=title, abstract, original title, name of substance word, subject heading word, keyword heading word, protocol supplementary concept word, rare disease supplementary concept word, unique identifier] (39374)

46 35 or 36 or 37 or 38 or 39 or 40 or 41 or 42 or 43 or 44 or 45 (482117)

47 exp Exercise Tolerance/ (7775)

48 exp Exercise Test/ (49008)

49 exercise.mp. [mp=title, abstract, original title, name of substance word, subject heading word, keyword heading word, protocol supplementary concept word, rare disease supplementary concept word, unique identifier] (226265)

50 exp Athletic Performance/ (37013)

51 performance.mp. [mp=title, abstract, original title, name of substance word, subject heading word, keyword heading word, protocol supplementary concept word, rare disease supplementary concept word, unique identifier] (572610)

52 exp Mountaineering/ (2191)

53 mountaineer*.mp. [mp=title, abstract, original title, name of substance word, subject heading word, keyword heading word, protocol supplementary concept word, rare disease supplementary concept word, unique identifier] (2459)

54 summit.mp. [mp=title, abstract, original title, name of substance word, subject heading word, keyword heading word, protocol supplementary concept word, rare disease supplementary concept word, unique identifier] (2589)

55 exp Physical Endurance/ (23651)

56 endurance.mp. [mp=title, abstract, original title, name of substance word, subject heading word, keyword heading word, protocol supplementary concept word, rare disease supplementary concept word, unique identifier] (25489)

57 47 or 48 or 49 or 50 or 51 or 52 or 53 or 54 or 55 or 56 (796079)

58 34 and 46 and 57 (123)

***************************
