# Supplementary material for: Genetic Factors Associated with Exercise Performance in Atmospheric Hypoxia
Source: Sports Med. 2015 Feb 15;45(5):745–61. doi: 10.1007/s40279-015-0309-8 (PMC4544548; doi:10.1007/s40279-015-0309-8)
Supplement: Supplementary file 2 — Supplementary material 2 (DOCX 12 kb) [file 40279_2015_309_MOESM2_ESM.docx]

Electronic Supplementary Material Appendix S2: Embase search strategy

Database: Embase Classic+Embase <1947 to 2014 January 31>

Search Strategy:

--------------------------------------------------------------------------------

1 exp Altitude/ (17323)

2 altitude.mp. [mp=title, abstract, subject headings, heading word, drug trade name, original title, device manufacturer, drug manufacturer, device trade name, keyword] (27944)

3 exp Anoxia/ (26762)

4 hypoxia.mp. [mp=title, abstract, subject headings, heading word, drug trade name, original title, device manufacturer, drug manufacturer, device trade name, keyword] (146973)

5 1 or 2 or 3 or 4 (182763)

6 exp Gene Frequency/ (85099)

7 (gene adj1 frequency).mp. [mp=title, abstract, subject headings, heading word, drug trade name, original title, device manufacturer, drug manufacturer, device trade name, keyword] (85933)

8 exp Genotype/ (278833)

9 genotype.mp. [mp=title, abstract, subject headings, heading word, drug trade name, original title, device manufacturer, drug manufacturer, device trade name, keyword] (284970)

10 exp Polymorphism, Genetic/ (302485)

11 polymorphism.mp. [mp=title, abstract, subject headings, heading word, drug trade name, original title, device manufacturer, drug manufacturer, device trade name, keyword] (305391)

12 exp Haplotypes/ (51522)

13 haplotype.mp. [mp=title, abstract, subject headings, heading word, drug trade name, original title, device manufacturer, drug manufacturer, device trade name, keyword] (62873)

14 (single adj1 nucleotide adj1 polymorphism).mp. [mp=title, abstract, subject headings, heading word, drug trade name, original title, device manufacturer, drug manufacturer, device trade name, keyword] (98174)

15 exp Genetic Linkage/ (67331)

16 (genetic adj1 linkage).mp. [mp=title, abstract, subject headings, heading word, drug trade name, original title, device manufacturer, drug manufacturer, device trade name, keyword] (46757)

17 6 or 7 or 8 or 9 or 10 or 11 or 12 or 13 or 14 or 15 or 16 (593734)

18 exp Exercise Tolerance/ (11543)

19 exp Exercise Test/ (44378)

20 exercise.mp. [mp=title, abstract, subject headings, heading word, drug trade name, original title, device manufacturer, drug manufacturer, device trade name, keyword] (348057)

21 exp Athletic Performance/ (4350)

22 performance.mp. [mp=title, abstract, subject headings, heading word, drug trade name, original title, device manufacturer, drug manufacturer, device trade name, keyword] (933298)

23 exp Mountaineering/ (2382)

24 mountaineer*.mp. [mp=title, abstract, subject headings, heading word, drug trade name, original title, device manufacturer, drug manufacturer, device trade name, keyword] (2694)

25 summit.mp. [mp=title, abstract, subject headings, heading word, drug trade name, original title, device manufacturer, drug manufacturer, device trade name, keyword] (3638)

26 exp Physical Endurance/ (16530)

27 endurance.mp. [mp=title, abstract, subject headings, heading word, drug trade name, original title, device manufacturer, drug manufacturer, device trade name, keyword] (30294)

28 18 or 19 or 20 or 21 or 22 or 23 or 24 or 25 or 26 or 27 (1257571)

29 5 and 17 and 28 (186)

30 exp Altitude/ (17323)

31 altitude.mp. [mp=title, abstract, subject headings, heading word, drug trade name, original title, device manufacturer, drug manufacturer, device trade name, keyword] (27944)

32 exp Anoxia/ (26762)

33 hypoxia.mp. [mp=title, abstract, subject headings, heading word, drug trade name, original title, device manufacturer, drug manufacturer, device trade name, keyword] (146973)

34 30 or 31 or 32 or 33 (182763)

35 exp Gene Frequency/ (85099)

36 (gene adj1 frequency).mp. [mp=title, abstract, subject headings, heading word, drug trade name, original title, device manufacturer, drug manufacturer, device trade name, keyword] (85933)

37 exp Genotype/ (278833)

38 genotype.mp. [mp=title, abstract, subject headings, heading word, drug trade name, original title, device manufacturer, drug manufacturer, device trade name, keyword] (284970)

39 exp Polymorphism, Genetic/ (302485)

40 polymorphism.mp. [mp=title, abstract, subject headings, heading word, drug trade name, original title, device manufacturer, drug manufacturer, device trade name, keyword] (305391)

41 exp Haplotypes/ (51522)

42 haplotype.mp. [mp=title, abstract, subject headings, heading word, drug trade name, original title, device manufacturer, drug manufacturer, device trade name, keyword] (62873)

43 (single adj1 nucleotide adj1 polymorphism).mp. [mp=title, abstract, subject headings, heading word, drug trade name, original title, device manufacturer, drug manufacturer, device trade name, keyword] (98174)

44 exp Genetic Linkage/ (67331)

45 (genetic adj1 linkage).mp. [mp=title, abstract, subject headings, heading word, drug trade name, original title, device manufacturer, drug manufacturer, device trade name, keyword] (46757)

46 35 or 36 or 37 or 38 or 39 or 40 or 41 or 42 or 43 or 44 or 45 (593734)

47 exp Exercise Tolerance/ (11543)

48 exp Exercise Test/ (44378)

49 exercise.mp. [mp=title, abstract, subject headings, heading word, drug trade name, original title, device manufacturer, drug manufacturer, device trade name, keyword] (348057)

50 exp Athletic Performance/ (4350)

51 performance.mp. [mp=title, abstract, subject headings, heading word, drug trade name, original title, device manufacturer, drug manufacturer, device trade name, keyword] (933298)

52 exp Mountaineering/ (2382)

53 mountaineer*.mp. [mp=title, abstract, subject headings, heading word, drug trade name, original title, device manufacturer, drug manufacturer, device trade name, keyword] (2694)

54 summit.mp. [mp=title, abstract, subject headings, heading word, drug trade name, original title, device manufacturer, drug manufacturer, device trade name, keyword] (3638)

55 exp Physical Endurance/ (16530)

56 endurance.mp. [mp=title, abstract, subject headings, heading word, drug trade name, original title, device manufacturer, drug manufacturer, device trade name, keyword] (30294)

57 47 or 48 or 49 or 50 or 51 or 52 or 53 or 54 or 55 or 56 (1257571)

58 34 and 46 and 57 (186)

59 exp altitude/ (17323)

60 altitude.mp. [mp=title, abstract, subject headings, heading word, drug trade name, original title, device manufacturer, drug manufacturer, device trade name, keyword] (27944)

61 exp hypoxia/ (79573)

62 hypoxia.mp. [mp=title, abstract, subject headings, heading word, drug trade name, original title, device manufacturer, drug manufacturer, device trade name, keyword] (146973)

63 59 or 60 or 61 or 62 (168307)

64 exp gene frequency/ (85099)

65 (gene adj1 frequency).mp. [mp=title, abstract, subject headings, heading word, drug trade name, original title, device manufacturer, drug manufacturer, device trade name, keyword] (85933)

66 exp genotype/ (278833)

67 genotype.mp. [mp=title, abstract, subject headings, heading word, drug trade name, original title, device manufacturer, drug manufacturer, device trade name, keyword] (284970)

68 exp genetic polymorphism/ (302485)

69 polymorphism.mp. [mp=title, abstract, subject headings, heading word, drug trade name, original title, device manufacturer, drug manufacturer, device trade name, keyword] (305391)

70 exp haplotype/ (51522)

71 haplotype.mp. [mp=title, abstract, subject headings, heading word, drug trade name, original title, device manufacturer, drug manufacturer, device trade name, keyword] (62873)

72 (single adj1 nucleotide adj1 polymorphism).mp. [mp=title, abstract, subject headings, heading word, drug trade name, original title, device manufacturer, drug manufacturer, device trade name, keyword] (98174)

73 exp genetic linkage/ (67331)

74 (genetic adj1 linkage).mp. [mp=title, abstract, subject headings, heading word, drug trade name, original title, device manufacturer, drug manufacturer, device trade name, keyword] (46757)

75 64 or 65 or 66 or 67 or 68 or 69 or 70 or 71 or 72 or 73 or 74 (593734)

76 exp exercise tolerance/ (11543)

77 exp exercise test/ (44378)

78 exercise.mp. [mp=title, abstract, subject headings, heading word, drug trade name, original title, device manufacturer, drug manufacturer, device trade name, keyword] (348057)

79 exp athletic performance/ (4350)

80 performance.mp. [mp=title, abstract, subject headings, heading word, drug trade name, original title, device manufacturer, drug manufacturer, device trade name, keyword] (933298)

81 exp mountaineering/ (2382)

82 mountaineer*.mp. [mp=title, abstract, subject headings, heading word, drug trade name, original title, device manufacturer, drug manufacturer, device trade name, keyword] (2694)

83 summit.mp. [mp=title, abstract, subject headings, heading word, drug trade name, original title, device manufacturer, drug manufacturer, device trade name, keyword] (3638)

84 exp endurance/ (16530)

85 endurance.mp. [mp=title, abstract, subject headings, heading word, drug trade name, original title, device manufacturer, drug manufacturer, device trade name, keyword] (30294)

86 76 or 77 or 78 or 79 or 80 or 81 or 82 or 83 or 84 or 85 (1257571)

87 63 and 75 and 86 (184)

***************************
